# “Twin-Chain” Hydrogels with Tailored Porosity, Surface Roughness, and Cleaning Capabilities

**DOI:** 10.1021/acs.langmuir.4c05381

**Published:** 2025-04-21

**Authors:** Rosangela Mastrangelo, Teresa Guaragnone, Andrea Casini, Damiano Bandelli, David Chelazzi, Piero Baglioni

**Affiliations:** † Center for Colloid and Surface Science, CSGI, via della Lastruccia, 3, Sesto Fiorentino, 50019 Florence, Italy; ‡ Department of Chemistry, University of Florence, via della Lastruccia, 3, Sesto Fiorentino, 50019 Florence, Italy

## Abstract

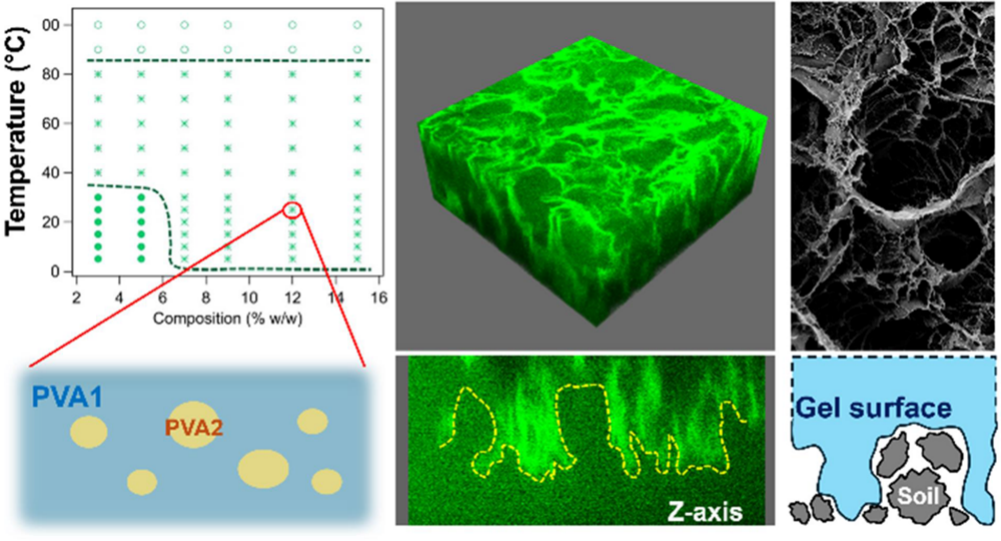

The remedial conservation of cultural heritage requires advanced functional materials that span from soft matter to nanostructured formulations, whose study is relevant to different fields, ranging from cosmetics and detergency to tissue engineering. In the vast landscape of innovative materials developed to counteract artwork degradation, gels have emerged as ideal candidates for cleaning delicate and complex surfaces. More specifically, twin-chain networks (TCNs), i.e., sponge-like hydrogels obtained through spontaneous polymer–polymer phase separation and sustainable freeze–thawing, showed unprecedented cleaning performances on modern/contemporary iconic paintings, such as works by Jackson Pollock and Pablo Picasso. The key to their efficacy lies in the complex cleaning mechanism at the gel–substrate interface. Recent findings suggest that the pore size and connectivity, affecting the nanoscale tortuosity, play a crucial role in determining the gels’ uptake (cleaning) ability. Nonetheless, diffusion of soil through the gel networks is only part of the complex processes that control the removal of unwanted layers from painted surfaces. A deeper understanding of the cleaning mechanism requires studying the uptake of solid particulate matter: gel adhesion and surface roughness affect the cleaning performance of a gel. In this work, TCNs with modulated porosity, obtained by mixing poly(vinyl alcohol)s (PVAs) of different hydrolysis degrees and molecular weights, were characterized through different techniques (confocal laser scanning microscopy, scanning electron microscopy, differential scanning calorimetry, and rheology) to relate porosity and structural features to the surface roughness and diffusional properties of the gels. Finally, cleaning tests on model painted surfaces revealed a clear connection between the surface inhomogeneity and the cleaning performance of the gels.

## Introduction

Research and industrial fields are increasingly exploring functional materials that combine high performances and innovation with sustainability, responding to pressing energetic and environmental requirements. In this context, the conservation of cultural heritage (CH) is a sector with high socioeconomic impact, where new materials are continuously needed and developed to counteract degradation processes exacerbated by climate changes and pollution.^1^ In addition to the importance of maintaining works of art to transfer them to future generations, the systems devised in this field, mainly belonging to soft matter and nanomaterials, hold potential applications in the food industry, cosmetics, detergency, tissue engineering, coatings, textiles, etc.^2−7^

In recent years, advanced materials for the remedial conservation of CH comprised soft matter systems, such as gels, and nanostructured cleaning fluids, tailored to the cleaning of sensitive surfaces as those of precious art masterpieces. Surface cleaning is a recurring and delicate task since dust, soil, and aged coatings, driven by environmental factors, pollution, or even serendipitous conservation interventions, can produce severe degradation and alteration at the surface of works of art and monuments. In such cases, cleaning or consolidation treatments are necessary to restore the original appearance of the artwork, avoiding damage to its original components. Gels, acting as scaffolds for cleaning fluids, and nanoparticles for consolidation, have been formulated over the years, recently including “green” synthetic, natural, and/or biodegradable polymers.^8−12^ In particular, among the wide range of gel matrices for the cleaning of painted surfaces, hydrogels show great effectiveness and versatility. These polymeric networks can embed water-based cleaning fluids for the removal of hydrophilic and even hydrophobic layers, with time and spatial control of the fluids’ release over surfaces. Namely, poly(vinyl alcohol) (PVA)-based “twin-chain networks” (TCNs), formed through a low-energy, sustainable freeze–thaw (FT) process, represent a novel class of highly effective hydrogel systems. These cryogels can be obtained through the combination of two PVAs with different hydrolysis degrees (HDs)^13,14^ or hydrophobicities,^15,16^ which spontaneously undergo phase separation in aqueous solution to create a sponge-like, interconnected porosity after FT. Highly hydrolyzed PVA (H-PVA), in excess in the formulation, concentrates in the aqueous continuous phase of the pregel, while partially hydrolyzed PVA (L-PVA), added in lower amounts, is dispersed and concentrates in blobs. The blobs act as templates during the FT process that allow the system gelation. The porogen L-PVA is partially extracted and removed during successive gel washing, leaving a micron-sized, interconnected porosity.^13−16^ Some L-PVA, however, remains adhered to the pore walls, conferring flexibility and compliance to mechanical stress.^13^ Overall, the process produces PVA TCNs with high elasticity, good flexibility, and water retention, which have proven to be ideal systems for the cleaning of textured and three-dimensional surfaces frequently found in modern/contemporary iconic paintings, such as the works of Jackson Pollock, Roy Lichtenstein, and Pablo Picasso.^13,17,18^

The micron-sized interconnected porosity and nanoscale tortuosity^14,16,19^ of TCNs impact the transport of simple and nanostructured fluids in the gel matrix,^20,21^ improving the cleaning abilities of the gels. In addition, a key feature is the size of pores, which directly affects the surface roughness of the gels^19^ and, indirectly, other crucial properties such as surface wettability, which affect the cleaning performances. Nanometric, submicrometric, and micrometric features at the gel surface determine macroscopic effects, such as adhesion to surfaces or surface tension.^22−24^ Moreover, surface roughness is also known to affect the sliding friction of gels on flat or jagged solid substrates.^25,26^ Yashima et al.^26^ found that gels with a rough surface (inhomogeneities of 2–20 μm) show significantly higher frictional stress than “flat-surface” gels. These authors also observed that the gel roughness enhances gel–substrate contact, while flat gels show larger areas with a thick water layer (several microns) at the gel–substrate interface, which weakens surface interactions and causes uneven adhesion of the gel to the surface. These reports show how crucial porosity and surface roughness are to diverse applications of gel networks, including the cleaning of CH, where gels must either adhere homogeneously to complex surfaces or work as “active erasers” by adhesion and absorption phenomena. Poor cleaning was indeed observed in the case of nonspongy PVA cryogels in contact with glass or paint surfaces.^13,14^ However, as crucial as porosity and surface roughness are, there is no thorough understanding of how these properties are affected by porogens in the FT gelation of the highly promising PVA TCNs, which is a gap considering their potential impact in transversal fields.

In this work, PVA-based TCNs were obtained using a series of PVA porogens with different molecular weights and HDs to systematically study, for the first time, the effect of the porogen on the porosity and surface roughness of the TCNs. Changes in the gel morphology at the microscale, as well as the rheology, crystallinity, and water release of the gels, were investigated. Porogen chain extraction after the FT process was monitored through NMR analysis. The porosity of the gels was related to their surface roughness at the microscale, and the ability of the gels to remove soil from paint mockups was assessed and related to the their roughness.

## Materials and Methods

### Chemicals

TCNs contain H-PVA (PVA with HD 98%) and the following L-PVA porogens: S88 (PVA with HD = 88% and shorter polymer chains), L88 (PVA with HD = 88% and longer polymer chains), S80 (PVA with HD = 80% and shorter polymer chains), and L80 (PVA with HD = 80% and longer polymer chains). All polymers were kindly supplied by Kuraray. The polymers were dissolved in microfiltrated water (Milli-Q), obtained through a Millipore system (resistivity >18 MΩ cm). The fluorescent dye Rhodamine 110 chloride (purity ≥99%, Sigma-Aldrich) was dissolved in water and used for confocal laser scanning microscopy (CLSM) gel imaging. Tribasic ammonium citrate (TAC, purity ≥97%, Sigma-Aldrich) was solubilized in Milli-Q water at a concentration of 5% w/w and loaded into the PVA-based gels to evaluate their cleaning performance.

### TCN Preparation

To formulate the TCNs, H-PVA was mixed with one of the porogen polymers (S88, L88, S80, or L80). A pure H-PVA hydrogel was also prepared and used as a reference (nonspongy) system. PVAs were dissolved in water, in a rounded-bottom flask immersed in an oil bath and equipped with a condenser, at 95 °C and under continuous stirring. After complete dissolution, polymer solutions were cooled down to 20 °C (4 h for S80 and L80 systems, 24h for S88 and L88 systems) and poured into polystyrene molds (14 × 7 × 0.2 cm^3^). Gelation was achieved through FT: the solutions were frozen at −18 °C (15 h) and then thawed, at room temperature, for 8 h. The process was repeated to obtain FT2 gels (i.e., 2 FT process were applied). Gels were finally washed in demineralized water for 7 days, changing the water daily, and stored in water. Gels containing the different porogens are indicated as G-S88, G-L88, G-S80, and G-L80, while G-H-PVA is the sample containing H-PVA only.

### Size Exclusion Chromatography

Size exclusion chromatography (SEC) measurements were performed utilizing a system composed of a Shodex ERC-3215α degasser connected with a Waters 1525 binary HPLC pump, a Waters 1500 series heater set at 50 °C, a Wyatt miniDAWN TREOS detector, a Wyatt Viscostar-II detector, a Wyatt OPTILAB T-rEX detector, a Shodex pre-column GPC KD-G 4A, and a Shodex column GPC KD-806 M employing a DMSO/DMF (70/30) mixture as the eluent with a flow rate of 0.65 or 0.70 mL min^–1^. Data evaluation was performed according to standard procedures employing ASTRA software.

### CLSM Imaging

The imaging of washed gels was performed using a Leica TCS SP8 confocal microscope (Leica Microsystems GmbH, Wetzlar, Germany) equipped with a 63X/1.2 Zeiss water immersion objective. Gels were previously soaked in a Rhodamine 110 chloride aqueous solution (24 h) to allow their diffusion throughout their volume. The dye was excited with an Ar ion laser (488 nm line), and fluorescence was collected with a photomultiplier tube in the 498–540 nm range. 3D images of the gels were obtained by stacking about 170 2D images.

### Scanning Electron Microscopy Imaging

Morphological analysis of the gels was performed using a SIGMA field-emission gun scanning electron microscope (FEG-SEM, Carl Zeiss Microscopy GmbH, Germany) with an acceleration potential of 2 kV and a working distance of 3.6 mm. Prior to scanning electron microscopy (SEM) analysis, freeze-dried gel samples were metallized with gold using an Agar Scientific Auto Sputter Coater.

### 3D CLSM Image Analysis

To estimate the average pore size, chord analysis distribution^27^ was implemented on 3D confocal images of the gels. The method allows one to calculate the characteristic dimensions of pores, λ. More in detail, we modified the MATLAB algorithm developed by MacIver and Pawlik^28^ to work on the Leica format (.lif), i.e., the 3D image format obtained directly from the microscope software. About 70 2D images of each stack (accounting for a thickness of ca. 35 μm) were binarized, and a set of 10,000 randomly oriented lines was drawn on them. Chords are extracted as segments, obtained when a line crosses phase boundaries (i.e., pore walls). The minimum chord length was set to 3 pixels (about 0.5 μm). Chords of specific lengths are binned according to their occurrence to finally plot their frequency, *f*(*R*), versus their dimension, *R* (μm). The trend for each sample is plotted as the average of the original data points. The persistence length λ is calculated as follows:
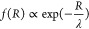
1

with 1/λ being the function slope in the semilog graph *f*(*R*)vs *R*. Uncertainties on data were set at the instrumental resolution.

### Gel Roughness

The gel roughness was calculated on black-and-white (binarized) confocal images of vertical cross sections of gels and by plotting their surface profiles using software ImageJ,^29^ according to a procedure previously reported.^19^ Intensity values were then normalized, and the roughness was calculated for each gel sample as the average normalized intensity, with the relative standard deviation.

### Release Experiments

To gain insights into the process of L-PVA release from the gels during washing steps, PVA TCNs were prepared as previously described via FT. After the thawing step, gel pieces of ∼ 1.0 × 1.0 × 0.2 cm^3^ were equilibrated in a 140 mM solution of acetone in 10 mL of D_2_O for 1 week (168 h), as previously reported for similar systems. During this time, gel samples were collected periodically and analyzed by means of proton nuclear magnetic resonance (^1^H NMR) to obtain the porogen mass release. ^1^H NMR spectra were measured at room temperature on a Bruker AVIII400 UltraShield Plus spectrometer. The residual ^1^H peak of the deuterated solvent (D_2_O) was used for chemical shift referencing. Calculations of porogen release were performed assuming that only L- or S-PVA was released for the gel samples containing porogens.

### Crystallinity Degree (*X*_c_ %)

The crystallinity degree of the TCNs was obtained through differential scanning calorimetry (DSC) measurements, performed on a Q2000 calorimeter (TA Instrument). The samples were subjected to a heating ramp in a nitrogen atmosphere, with a flow rate of 50 mL/min, using a heating rate of 5 °C/min. The temperature range was set from 25 to 250 °C. The melting peaks observed for the freeze-dried samples were integrated through the TA TRIOS software. *X*_c_ was calculated as the ratio between the specific enthalpies of fusion of each sample and a fully crystalline PVA (161 J/g),^30^ respectively. *X*_c_ values are reported as averages of three measurements.

### Gel Fraction (*G*%)

The fraction of insoluble polymer chains in the gels was evaluated gravimetrically. More specifically, the gel fraction was obtained as follow:
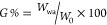
2

where *W*_0_ is the dry gel weight before washing, and *W*_wa_ is the dry gel weight after washing. *W*_wa_ was obtained by oven drying the TCNs and G-H-PVA at 120 °C, until reaching a constant weight. *G*(%) values are reported as averages of three measurements.

### Water Release

After the removal of excess water from the surface of washed gels, 2.5 × 2.5 × 0.2 cm^3^ gel sheets were gently placed on Whatman filter paper in a Petri dish and covered with a lid to limit evaporation. The weight of the water absorbed by the paper was recorded after 15 min. The values reported are averages of three measurements.

### Rheology

Rheological measurements were performed with a Discovery HR-3 rheometer from TA Instruments (40 mm diameter parallel plate geometry). Amplitude sweeps were collected at a constant frequency (1 Hz) to identify the linear viscoelastic range. Frequency sweeps were then collected at 0.08% amplitude for all gel samples. The temperature was controlled through a Peltier element and held at 25 °C during the measurements. Data reported are averages of three repetitions obtained by testing different areas of each gel sample.

### Paint Mock-Up Preparation

A white acrylic mock-up panel paint was obtained by spray-coating a plain plywood with a matte acrylic paint (Spray Montana MTN94, Montana Colors). The painted surface was left to cure at room temperature for 7 days to ensure full drying. Then, to simulate realistic soiling, the surfaces were treated with a mixture comprising organic and inorganic dust particles combined with oils and suspended in nonane, following an established artificial soiling protocol for paints.^13^

### Cleaning Test

To evaluate the cleaning efficacy of the gels, the different gel formulations (see TCN Preparation) were loaded with an aqueous solution of TAC (5 wt %). The cleaning trials involved direct application of the gels onto the artificially soiled white acrylic mock-up. Prior to use, gel sheets (2 × 2 × 0.2 cm^3^) were gently blotted with paper to remove excess water from their surface. The gels were then applied to soiled areas for 10 min. Then, the gels were carefully lifted using tweezers to avoid any additional mechanical action on the paint surface. The treated paint surfaces were allowed to dry without any further manipulation (no rinsing), to allow unbiased evaluation of the soil uptake capacity of each gel system in a single application. Each gel type was tested in triplicate, each time on a different section of the panel.

The cleaning efficacy was evaluated through a semiquantitative approach, adapted from the protocol established by Casini et al.^15,31^ High-resolution images of the treated surfaces were captured using a Canon EOS 5D Mark II DSLR camera coupled with a Sigma 105 mm f/2.8 EX macro lens. To mitigate shadowing, the lighting was positioned at 45° angles on both sides of the sample. The captured images underwent geometric correction using GNU Image Manipulation Program (GIMP), followed by detailed analysis with ImageJ software.^29^ For each treated paint area, a central section of 1.5 × 1.5 cm^2^ was selected, to exclude edge movement of soil originated when lifting the gels with tweezers. Subsequently, the images were converted to the grayscale and segmented using 17 distinct thresholding algorithms available within ImageJ. Each image was partitioned into 72 × 72-pixel segments, with average grayscale values being compared to the corresponding areas in the original 8-bit images. Only the thresholding methods that produced higher correlation coefficients (*R*^2^) were retained for further analysis. The cleaning performance was then calculated as the percentage by normalizing the decrease in black pixels of the soiled surface after cleaning and subtracting this value from 100. The values are reported as averages with their standard deviations.

### Fourier Transform Infrared Spectroscopy

Micron-scale Fourier transform infrared (FTIR) 2D imaging was carried out on the treated areas of the artificially soiled acrylic mock-up, using a Cary 620–670 FTIR microscope (Agilent Technologies) equipped with a 128 × 128 focal plane array (FPA) detector. Spectra were recorded directly on the sample surface in reflectance mode with an open aperture, with a spectral resolution of 8 cm^–1^ and acquiring 128 scans per spectrum. A single “tile″ map, with dimensions of 700 × 700 μm^2^ and a pixel size of 5.5 × 5.5 μm^2^, was used for analysis, where each pixel yields an independent spectrum. The intensity of characteristic bands was imaged across the 2D maps, with the false color chromatic scale indicating increasing absorbance from blue (lowest) to red (highest). A representative surface map of approximately 1.4 × 2.1 mm (i.e. a mosaic of 2 × 3 tiles) was acquired on each of the gel-treated paint areas for direct comparison.

## Results and Discussion

### Polymer Characterization and Phase Behavior

Our recent studies on TCN sponge-like cryogels emphasized the importance of polymer conformation and phase behavior in solution as key aspects in determining the final gel properties. Polymer–water and polymer–polymer interactions determine phase separations, influencing the size of micron-sized domains formed in pregel aqueous solutions.^13−16,19^ More specifically, TCNs are a class of PVA cryogels where the formation of micron-sized pores is granted by PVA liquid–liquid phase separation: the porogen polymers, featuring an HD between 80 and 88%, spontaneously undergo phase separation in the pregel solution, templating the morphology of the gels during and after the FT cycles.

In this work, PVA pairs were mixed to favor the formation of sponge-like gels with a modulated pore size, affecting both diffusional properties and the surface roughness of the gel networks.

The SEC analysis of the highly hydrolyzed PVA (H-PVA) revealed a number-average molar mass (*M*_n_) of 100 kg mol^–1^ and a dispersity index (*Đ*) of 1.38 (Table 1). Higher *M*_n_ values were obtained for the L-PVA series, ranging between 143 and 203 kg mol^–1^, while *Đ* values ranged between 1.35 and 1.65.

**Table 1:** Parameters Obtained by SEC Analysis of the PVAs Used for the Preparation of TCNs

polymer	HD [%]	*M*_n_ [kg mol^–1^]	*Đ*	*R*_h_^a^ [nm]	*a*_MH_^a^
H-PVA	>98	100 ± 1	1.38 ± 0.01	10.9	0.56
S88	88	143 ± 1	1.65 ± 0.02	9.7	0.64
L88	88	183 ± 1	1.58 ± 0.01	11.1	0.67
S80	80	154 ± 1	1.51 ± 0.01	10.3	0.68
L80	80	203 ± 1	1.35 ± 0.01	13.9	0.61

a Errors are below the last decimal reported.

**Figure 1:**
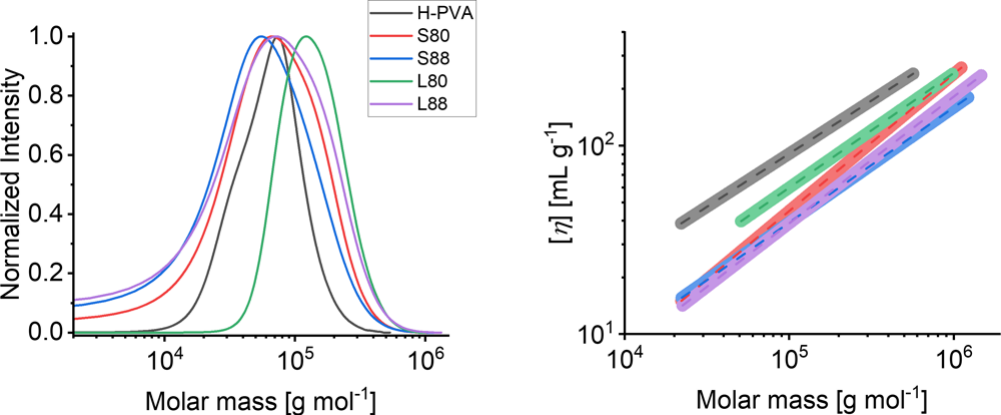
SEC analysis of the PVA samples. Left: number-average molar mass distributions. Right: Mark–Houwink plots of the intrinsic viscosity [η] vs molar mass of the polymers.

Given the moderate molar mass dispersity of each PVA sample, the estimation of the molar mass distributions is of outmost importance to decouple the effects of high and low molar mass tailing during the preparation of FT samples. In this regard, the fully hydrolyzed H-PVA (*Đ* = 1.38) exhibited molar mass ranging between 10^4^ and 5 × 10^5^ g mol^–1^. Moreover, despite having different *M*_n_ values, the partially hydrolyzed S80, S88, and L88 featured similar molar mass distributions in the range of 2 × 10^3^–10^6^ g mol^–1^, while L80 exhibited higher molar mass (2 × 10^4^–10^6^ g mol^–1^, Figure 1).

Besides, both H- and L-PVAs exhibited different hydrodynamic radii (*R*_h_) in the hydrophilic DMSO/DMF (70/30 v%) mobile phase. In particular, L-PVAs S80, S88, and L88, sharing similar molar mass distributions, featured different *R*_h_ values, indicating variations of chain extension in the selected solvent mixture. To gain additional information on this behavior, the intrinsic viscosity [η] of the polymers, i.e., the viscosity of a solution ideally containing one macromolecule, was plotted vs their molar mass [*M*], according to the Mark–Houwink equation (eq 3, Figure 1).
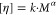
3

where *k* and α are the Mark–Houwink parameters describing polymer–solvent interactions and, thus, the PVA morphology in solution. Briefly, α values range between 0.50 and 0.78, indicating favorable polymer–solvent regimes.^9^ Overall, the obtained data confirmed good solvent behavior for all of the selected PVAs in DMSO/DMF (70/30 v%), in line with the recent literature.^15,16^

As mentioned above, sponge-like gels can be obtained by mixing H-PVA with one of the porogens in an aqueous solution. To elucidate the polymer behavior in water, the phase diagrams of each polymer in aqueous solution were investigated (Figure S1). H-PVA is highly hydrolyzed, with less than 2% residual acetate groups (Table 1). Due to the high number of −OH groups, interaction with water is favored by hydrogen bonding, and H-PVA aqueous solutions are transparent and homogeneous in the investigated range of temperatures and concentrations (Figure S1A). The porogen PVA solutions, on the other hand, are characterized by a lower critical solution temperature (LCST) (Figure S1B–E).^13,32−34^ The LCST is characteristic of partially hydrolyzed PVAs, where residual acetate groups impart a hydrophobic character to the polymer chains.^35^ The molecular weight and blockiness also play roles in determining polymer solubility and the onset of the cloud point in solution.^34,36,37^ In this case, S88 and L88 show a cloud point around 85 °C at high polymer concentrations, which reduces to 60 °C for S80 and L80, due to the lower HD. The effect of the polymer chain length is visible at lower polymer concentrations in the diagrams: lower-molecular-weight polymers show reduced or absent cloud point areas at concentrations of 3–7% w/w (Figure S1B,D).

**Figure 2:**
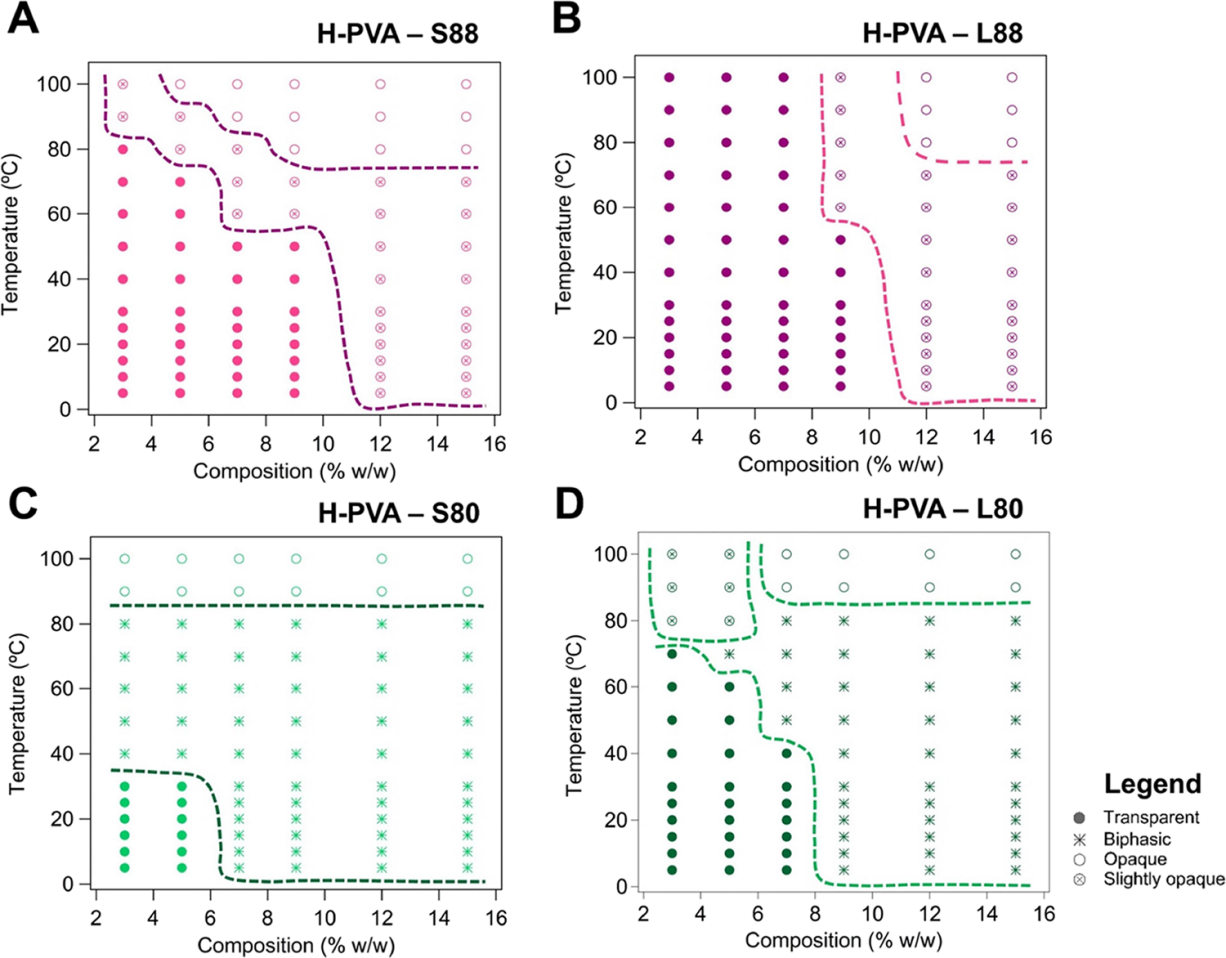
Phase behavior of H-PVA–L-PVA mixtures in aqueous solution: (A) H-PVA–S-88, (B) H-PVA–L88, (C) H-PVA–S80, (D) H-PVA–L80. Dashed lines indicate the different phases of the diagram and have been included to enhance the image readability.

The phase diagrams of H-PVA–L-PVA mixtures (3:1 ratio as in gel formulations) show a more complex scenario (Figure 2), where the *M*_n_ and HD of L-PVAs are keys to determine the polymer miscibility at different temperatures.

More specifically, in this case, both polymer–water (linked to LCST) and polymer–polymer phase separation are expected to occur contextually.^13,32^ The LCST behavior is evident in all systems at temperatures higher than 40–60 °C. In H-PVA–S88 and H-PVA–L88 mixtures (Figure 2A,B), polymer–polymer phase separation can be inferred in opalescent solutions, even at lower temperatures than the LCST, especially at polymer concentration >12% w/w.

Mixtures containing S80 and L80, on the other hand, undergo macroscopic polymer–polymer phase separation upon cooling (Figure 2C,D); such phase separation appears within 24 h from polymer dissolution.

Since H-PVA–L-PVA demixing occurs through the formation and growth of micron-sized L-PVA domains,^13,14^ pregel solutions were stored at 25 °C for different times to obtain TCNs with different pore sizes. More specifically, considering that S80 and L80 formulations showed macroscopic phase separation within 24 h from dissolution, solution storage lasted only 4 h in this case. S88 and L88 formulations were stored at 25 °C for 24 h, as their phase behavior involved a slower phase separation, similar to other systems previously investigated.^13,14,19^ After storing, all systems underwent two FT cycles to induce gelation. Porous gels were obtained after repeated washing steps in demineralized water.

### Gel Morphology at the Micro- and Submicron Scale

**Figure 3:**
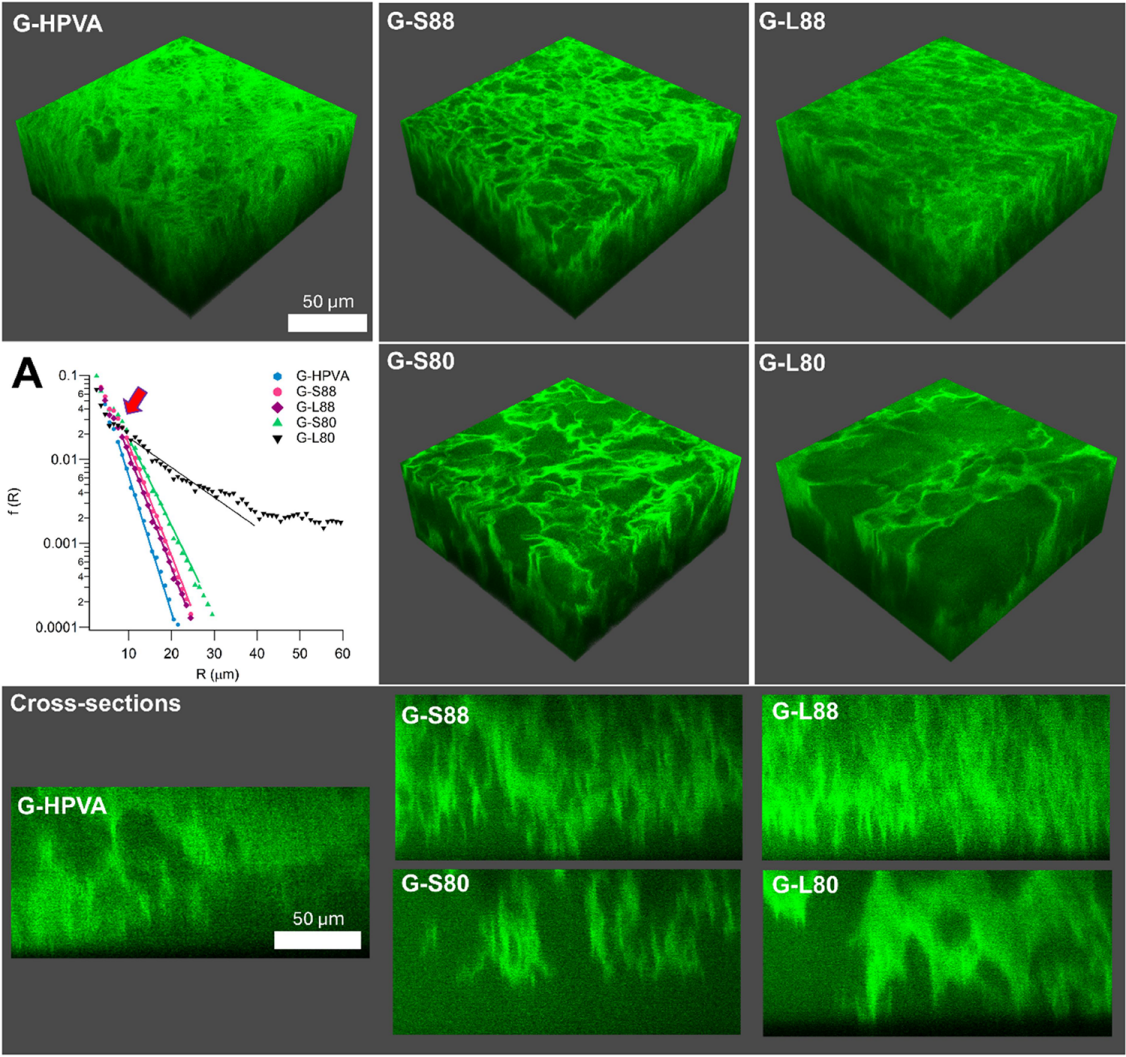
3D laser confocal images and cross sections of the investigated gels. (A) Chord length analysis plot.

The microscale morphology of gels after washing and storage in water was observed by CLSM. 3D images of H-PVA gel (G-HPVA) and the TCNs are shown in Figure 3. H-PVA clearly shows smaller and more isolated pores, while TCNs feature a sponge-like, interconnected porosity. The pore size is expected to be dependent on the HD and *M*_n_ of the porogens. More specifically, longer and more hydrophobic L-PVA chains are expected to form larger blobs in the pregel solutions, leading to larger gel pores.^16,19^ In this case, the effect of HD is much larger than that of *M*_n_. The characteristic length of pores, λ, ranges from 3 to 12 μm (Table 2). The calculation of the persistence length λ of the pore phase was performed through the chord analysis method,^38,38^ applied on binarized 3D confocal stacks. The frequency of chords, *f*(*R*), vs their size *R* is shown in Figure 3, panel A. λ, the characteristic pore size, is obtained as the inverse of the curves slopes,^39^ after the short plateau, indicating similar recurrent pores size in all TCNs (7.5–8.5 μm; see arrow in Figure 3, panel A). However, λ describes only an average dimension, and pore size spans up to 20–30 μm in all samples, except G-L80, where pores up to 100 μm were observed.

**Table 2:** Average Pore Size (from CLSM Images), Surface Roughness (Obtained as the Average Intensity of Cross Sections Profiles and the Relative Standard Deviations), Crystallinity *X*_c_ (%), Crystallite Melting Temperature *T*_m_, Gel Fraction *G* (%), Storage Modulus at 1 Hz, *G*′ (1 Hz), and Water Release of the Gels

gel	λ (μm), ±0.3	surface roughness (%)	*X*_c_ (%)	*T*_m_ (°C)	*G* (%)	*G*′ at 1 Hz (kPa)	water release (mg/cm^2^)
G-H-PVA	2.7	28 ± 16	27 ± 1	207 ± 1	58 ± 3	2.3	27 ± 1
G-S88	3.3	20 ± 11	28 ± 2	210 ± 2	39 ± 2	3.2	26 ± 1
G-L88	3.2	9 ± 5	27 ± 4	209 ± 3	38 ± 2	2.8	23 ± 1
G-S80	4.2	55 ± 26	31 ± 2	212 ± 2	46 ± 2	5.8	21 ± 2
G-L80	12.2	45 ± 26	24 ± 3	216 ± 5	47 ± 1	4.4	22 ± 1

Therefore, it can be concluded that the pore size follows the trend G-L80 > G-S80 > G-L88 = G-S88.

Pore sizes can be related to the surface roughness of the gels, as larger pores determine the formation of deeper valleys (rugosity) at the gel surface. Confocal images of the vertical cross sections of the gels are shown in Figure 3. A profile was extracted from the latter, and the roughness was calculated as the average of the normalized profile intensity with the relative standard deviation (see Table 2). G-80 shows the most irregular profiles, inferred from higher intensities and larger standard deviations. This is expected to facilitate solid particle uptake from surfaces during cleaning operations.^19^

Porosity in the TCNs is hierarchical,^13,14^ extending across the micro- and submicron scale, and affecting transport properties,^14,16,21^ which are crucial in surface cleaning.

**Figure 4:**
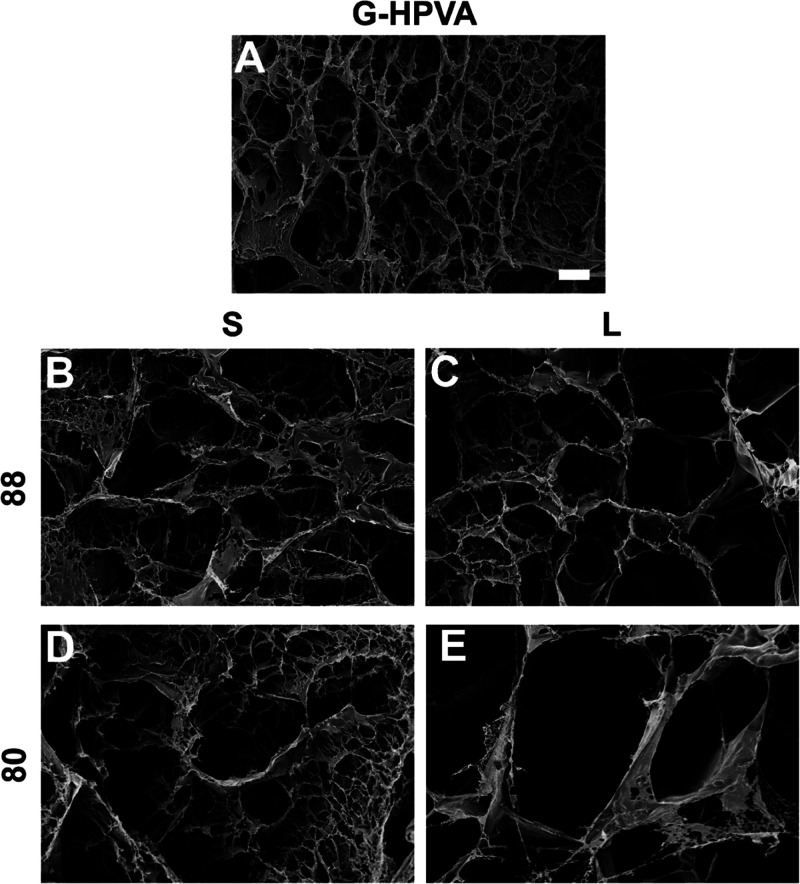
SEM micrographs of the investigated gels: (A) G-HPVA, (B) G-S88, (C) G-L88, (D) G-S80, and (E) G-L80. Scale bar: 5 μm.

The SEM images collected on freeze-dried gels show macropores compatible with those characterized by CLSM and indicate the presence of submicron pore architectures (Figure 4). The largest pores of G-S80 and G-L80 can be related to those observed through confocal microscopy. The widest pore range is observable in G-S80, with many pores as small as 0.5 μm. Nonetheless, submicron pores were visible in all samples except G-L80.

Overall, CLSM and SEM micrographs, together with the observed conformational and phase behavior of the polymers, suggest that long-chain L-PVAs with a more hydrophobic character, such as L80, are more prone to phase separate and form large, micron-sized pores. It is actually known that polymer pairs undergo phase separation more easily as their molecular weight increases,^40^ and phase separation has been exploited to obtain gels with wide pores, both PVA-based^41−44^ and from different macromolecules.^45−47^

### Gel Structure and Mechanical Properties

The FT process, repeated twice on the H-PVA–L-PVA pregel solutions, determines the formation of physical, 3D networks. PVA gelation at low temperature occurs due to a water–polymer phase separation, leading to the formation of water-rich and polymer-rich areas. Eventually, ice forms and the pressure over the polymer-rich pools causes the formation of PVA crystallites. The crystallinity of the final networks depends on the polymer concentration, the number of FT cycles, and the presence of polymer–polymer phase separation.^48−50^ The latter can influence the size of crystalline domains as the presence of L-PVA blobs causes a local increase in the H-PVA concentration in the continuous phase.

Moreover, repeated FT cycles are expected to affect the gel fraction, i.e., the fraction of polymer chains actively included in the gel network; namely, the gel fraction accounts for the polymer chains retained within the gel after washing, i.e., chains interacting with each other, through hydrogen bonding, entanglements, or included in crystallites.

The cryo-structuration of gels occurring during freezing also has an effect on its rheological properties. More specifically, repeated FT cycles and high local polymer concentrations lead to stiffer gels, due not only to the formation of polymer crystallites but also to the formation of structural chain entanglement in the networks.

**Figure 5:**
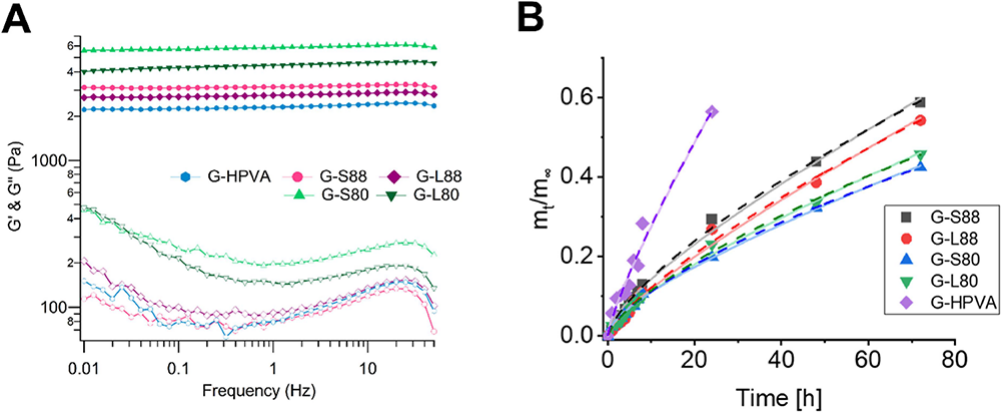
(A) Frequency sweeps of gels and (B) release of PVA from the gels. Left: relative mass release vs time, and fitting to the Peppas–Korsmeyer (lines, eq 4) and Peppas–Salhin (dotted lines, eq 5) models. Errors are included in the markers.

The gel crystallinity *X*_c_ (%) and crystallites average melting temperature *T*_m_, obtained by DSC measurements, and the gel fraction *G* (%) and storage modulus at 1 Hz *G*′ (1 Hz), measured through rheometry, are presented in Table 2. The frequency sweeps of the gels are reported in Figure 5A.

The interpretation of the physicochemical parameters reported in Table 2 is not straightforward. Overall, *X*_c_ and *T*_m_ do not vary significantly along the series, but important trends emerge. The crystallinity of the gels is the same for G-HPVA and G-88, while that of G-80 samples is significantly different, being S80 as the most crystalline network. Moreover, *T*_m_ indicates that stable crystallites form in G-L80, the gel with the lowest crystallinity.

The gel fraction indicates that the addition of L-PVA causes, in general, a larger loss of polymer chains after washing; G-80 and G-88 *G* (%) values are significantly different: the stronger polymer–polymer phase separation in G-80 gels probably leads to a neat increase in the H-PVA local concentration in the continuous phase. This should, in general, increase the crystallinity of the gel. Nonetheless, the different *M*_n_ values of the porogens produce different results: higher crystallinity was measured in G-S80, as S80 chains likely do not affect the cryo-structuration process, being only slightly longer than H-PVA chains. Conversely, L80 chains are long enough to disrupt H-PVA crystallization during freezing: in this case, denser crystallites form, but the crystalline portions are accordingly smaller.

Longer polymer chains and sharper phase separations are more likely to cause the formation of entanglements during freezing, producing networks with high elasticity, independent of the crystallinity degree.^14^ This is the case of G-80 samples, characterized by a significantly higher elasticity (higher *G*′ in Figure 5A and Table 2) compared to the other networks.

### Porogen Release Study

As mentioned above, the overall gel porosity affects diffusional and transport properties within the gel matrix. Polymer chain diffusion through the gels during washing was investigated by NMR spectroscopy. More in detail, the release of PVA chains during washing steps was studied in D_2_O to gain information on the porogen release from the gels (see Materials and Methods). To this aim, the four gels containing L-PVA porogens and a blank gel (G-HPVA), were equilibrated in a diluted acetone solution in D_2_O for 1 week (168 h). As expected, ^1^H NMR analysis performed on solution aliquots, collected at different equilibration times, showed the release of PVA species from the gels. However, the blank gel also releases PVA, likely low molar mass fractions of H-PVA that were not detected when fully hydrolyzed PVA with a higher molar mass was employed for the gel preparation.^15^

Plots of the release fraction vs time were fitted to the Peppas–Korsmeyer equation (eq 4).
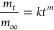
4

where *m_t_*/*m*_∞_ is the mass release fraction at time *t*, *k* is a scale factor, and *m* is a shape factor correlated to the release process. Briefly, *m* values ≤0.5 indicate a purely Fickian PVA release, *m* ≥ 1 indicate case II transport (i.e., swelling of polymer chains), and values between the two regimes indicate a balance between diffusion and swelling. Fittings were performed for data points up to 72 h of swelling, corresponding to a mass release fraction <0.6, as required by the model.

In all cases, *m* values ranged from 0.7 to 0.9, suggesting that both diffusion and swelling of the polymer chains occur during the polymer release (Figure 5B, Table 3).

**Table 3:** Parameters Obtained from Fitting the Porogen Release of the G-S, G-L, and G-HPVA Gels to the Peppas–Korsmeyer (P–K) and Peppas–Salhin (P–S) Equations

code	*m* (P–K)	*R*^2^ (P–K)	*k*_F_ (P–S)	*k*_R_ (P–S)	*R*^2^ (P–S)
G-HPVA	0.82 ± 0.06	0.970	0.021 ± 0.011	0.026 ± 0.004	0.974
G-S88	0.71 ± 0.02	0.998	0.023 ± 0.002	0.005 ± 0.003	0.998
G-L88	0.76 ± 0.03	0.993	0.017 ± 0.004	0.008 ± 0.001	0.991
G-S80	0.67 ± 0.01	0.999	0.026 ± 0.003	0.008 ± 0.001	0.995
G-L80	0.70 ± 0.02	0.997	0.021 ± 0.003	0.006 ± 0.001	0.995

Additional information was provided by fitting the release data to the Peppas–Salhin equation, which enables one to decouple the effect of Fickian and relaxation (swelling) processes (eq 5).
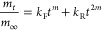
5

where *k*_F_ and *k*_R_ are constants related to the Fickian and relaxation processes, respectively. In this model, the *m* parameter is a purely Fickian diffusion coefficient whose value depends on the aspect ratio of the sample (2*a*/*l*, where *a* is the diameter and *l* the thickness of the releasing matrix^51,52^), which was 0.46 in the case of the tested gels.

The fittings were fully consistent with those obtained using eq 4 (see Figure 5B), and G-HPVA showed the highest release rate. It is also worth noting that *k*_R_ is an order of magnitude lower than *k*_F_ in all TCNs, suggesting that diffusion-controlled polymer release becomes prevalent when porogens are added to the formulation. The decreased release capability of the additive-containing gels could be related to their ability to “force” H-PVA in denser packing, which in turn varies with the molar mass and degree of hydrolysis of the porogens. Finally, *k*_F_ is slightly higher than in other samples. As the SEM imaging suggested a higher polydispersity in the porosity of G-S80, this might result in a more pronounced diffusion-controlled release in this gel.

### TCN Cleaning Assessment

The cleaning efficacy of the gel was assessed on a white acrylic paint mock-up, artificially soiled, as detailed in the Materials and Methods section, to evaluate their performance on flat and minimally textured surfaces.

**Figure 6:**
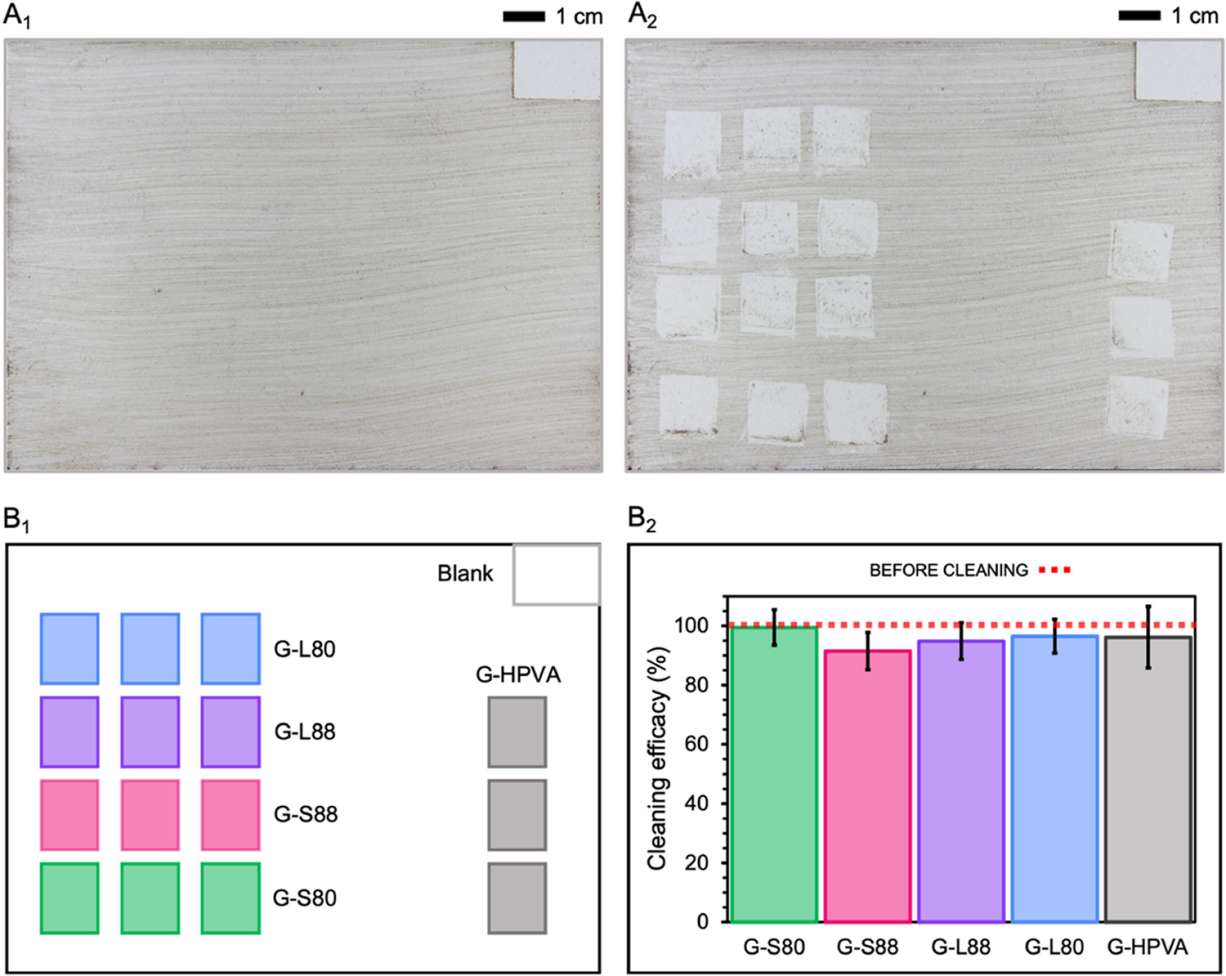
Macro-images of the soiled white acrylic mock-up surface (A_1_) before and (A_2_) after treatment with G-S80, G-S88, G-L88, G-L80, and G-HPVA gel systems loaded with 5 wt % aqueous TAC solution. (B_1_) Keys of cleaning spots of the different gel systems and the pristine acrylic white paint (Blank). (B_2_) Soil removal efficiency, expressed as the percentage of nonsoiled pixels normalized to the soiled surface (before cleaning), for each gel system after a single cleaning step (10 min without rinsing), as determined through a semiquantitative image analysis.

As shown in Figure 6, the G-S80 gel formulation exhibits the highest average cleaning efficiency after a single application, achieving nearly complete soil removal (≈100%). In contrast, G-L88, G-L80, and G-S88 display lower cleaning performances (≈90–95%). The G-HPVA gel, composed solely of H-PVA, shows an inconsistent cleaning efficacy with high variability across three independent areas, resulting in the largest standard deviation observed. This result is likely due to its macroscopically heterogeneous structure, which produces significant differences in the cleaning performance over different spots of the same treated area.

**Figure 7:**
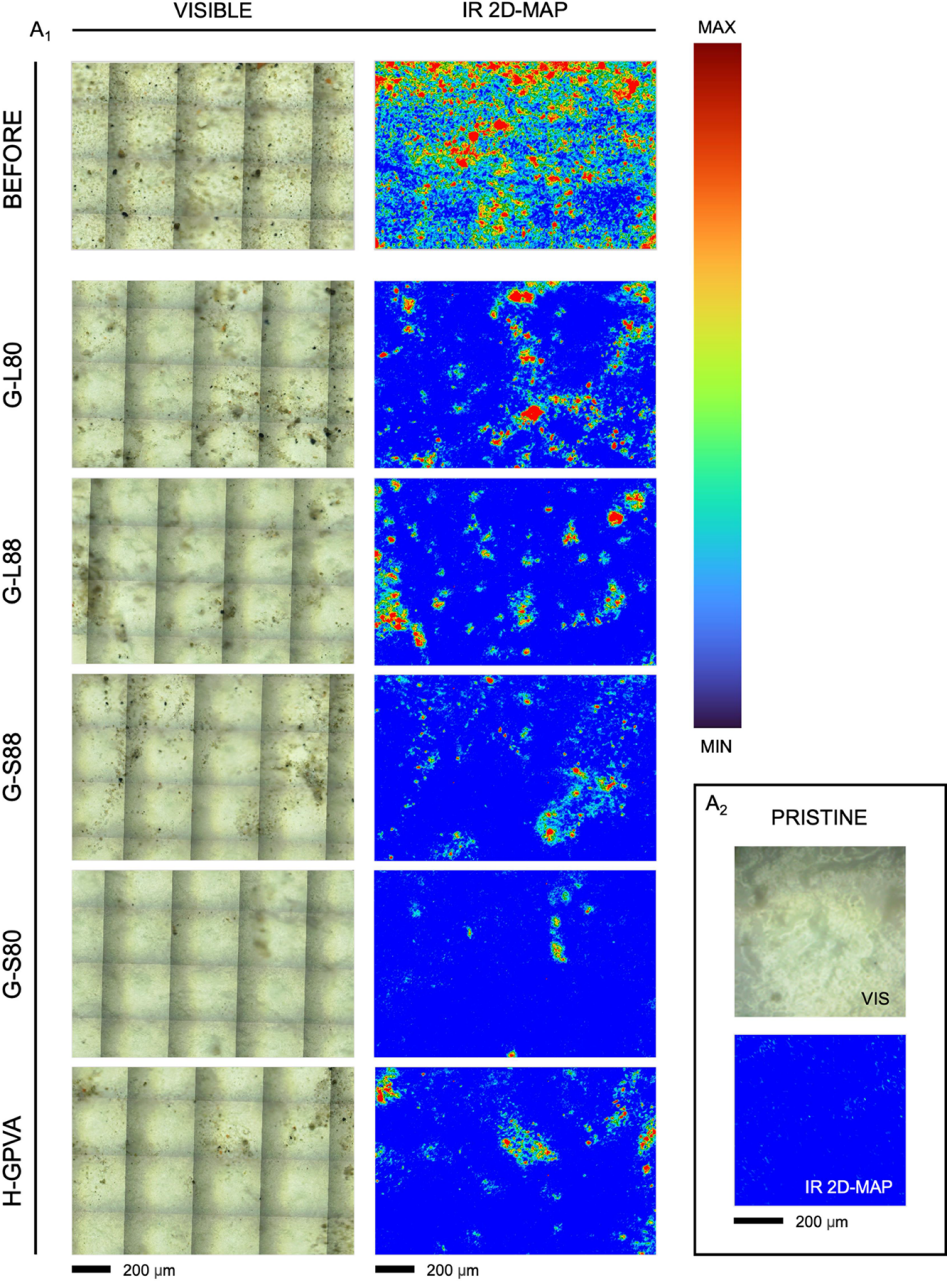
FTIR 2D imaging of the white acrylic mock-up: pristine (A_2_ panel), soiled (BEFORE), and after one cleaning step (10 min without rinsing) using G-S80, G-S88, G-L88, G-L80, and H-GPVA gels loaded with 5 wt % aqueous TAC solution (A_1_ panel). Adjacent to each visible map, the corresponding 2D FTIR false color map illustrates the intensity of the hydroxyl stretching bands of kaolin (3725–3595 cm^–1^) in the artificial soil.

Even though they are not directly comparable to the macro-images shown in Figure 6, owing to the higher detection limit of the FPA detector (∼0.02 pg/μm^2^),^13^ the FTIR 2D maps confirmed the higher efficacy of G-S80 in soil removal by uptake in a single application. Mapping the OH-stretching bands of kaolin (present in the soil) in the 3725–3592 cm^–1^ range demonstrated the extent of soil removal at the micron scale (Figures 7 and S2). It is evident that G-S80 produced the best cleaning result even at this scale. The other gels yielded partially cleaned microareas, whose variability falls within the deviations observed at the macroscopic scale through imaging.

Importantly, no detectable PVA residues were observed on the treated surfaces (Figures S3 and S2), down to the detection limit of the FPA detector, in agreement with previous studies.^13^

Several critical factors contribute to the optimal cleaning performance of G-S80. Its heterogeneous porosity increases the diffusion-controlled transport within the matrix and produces increased surface roughness, enhancing superficial adhesion, while its interconnected pores (a unique feature of TCNs versus the H-PVA gel) favor the capture and uptake of soil particles. In addition, the lower elastic modulus of the gel allows better compliance and adhesion to surfaces, granting homogeneous cleaning.

## Conclusions

In this work, TCN hydrogels with modulated porosity for enhanced soil uptake were obtained by combining PVAs with different HDs and molecular weights (*M*_n_). More in detail, shorter (S)- and longer (L)-chain PVAs, with HD = 88 or 80%, were mixed with a highly hydrolyzed PVA (H-PVA, HD = 98%). The polymer phase behavior in water and in pregel solutions suggested stronger phase separations when the HD of the additive differs the most from H-PVA. All of the obtained TCNs show higher porosity than the pure G-HPVA gel, with the average pore size increasing as the HD of the L-PVAs decreases (i.e., as phase separation becomes more pronounced). The surface roughness of the gels could be partially related to the morphology and pore size, with an increase in polydispersity in systems with intermediate *M*_n_ porogens. The crystallinity and mechanical properties of the gels confirmed that different phase separation behaviors occur in pregel solutions as the HD is changed, affecting the structural features of the final gel. Diffusional properties of the gels, tested through NMR spectroscopy and specifically monitoring polymer chain extraction during washing, suggest that diffusion-controlled polymer release is prevalent in all TCNs, while G-HPVA shows comparable swelling and diffusional dynamics. G-S80 emerges as the sample with the strongest diffusional-controlled behavior; while its microscale porosity is intermediate (average pore size of ca. 4 μm), it shows a higher pore polydispersity in SEM micrographs and the highest surface roughness. Indeed, this gel formulation showed the most effective soil removal from a painted surface, owing to the combination of optimized surface roughness, adhesion, porosity, and overall gel elasticity. It is worth noting that this is the first case where we observed a lower cleaning performance for the largest-pore gel (G-L80): in all of our previous studies, larger pores were probably related to higher polydispersity, resulting in higher network tortuosity and cleaning ability. These findings suggest that the pore polydispersity might be a crucial factor to consider when evaluating the cleaning ability of hydrogels. Overall, we showed how the porosity and structural features of PVA TCNs can be controlled and related to the surface roughness and diffusional properties of the gels, playing on polymer–polymer phase separation driven by changes in the HD in pregel solutions. The clear connection we highlighted between the surface inhomogeneity and cleaning performance of the gels opens a pathway for the formulation of hydrogels with enhanced cleaning and detergency capacity to be used not only in the preservation of iconic CH objects but also in other sectors such as cosmetics, tissue engineering, coatings, food industry, etc.

## Supporting Information Available

Additional figures (Figures S1–S3), showing the phase diagrams of pure polymers in aqueous solution, FTIR spectra, and FTIR 2D images of the substrate used for the cleaning tests; additional tables (Table S1), listing the 1/λ values (chord-length distribution) (PDF)
