# Supplementary material for: “Twin-Chain” Hydrogels with Tailored Porosity, Surface Roughness, and Cleaning Capabilities
Source: Langmuir. 2025 Apr 21;41(16):10238–49. doi: 10.1021/acs.langmuir.4c05381 (PMC12044679; doi:10.1021/acs.langmuir.4c05381)
Supplement: Supplementary file 1 — la4c05381_si_001.pdf [file la4c05381_si_001.pdf]

Supporting information for

“Twin-Chain” hydrogels with tailored porosity, surface roughness and cleaning capabilities

R. Mastrangelo[a], T. Guaragnone[a], A. Casini[a], D. Bandelli[b], D. Chelazzi[a,b] and P. Baglioni[a*]*

[a] *Center for Colloid and Surface Science, CSGI, via della Lastruccia, 3, Sesto Fiorentino, Florence, 50019, Italy*

[b] *Department of Chemistry , University of Florence, via della Lastruccia, 3, Sesto Fiorentino, Florence, 50019, Italy*

KEYWORDS (Word Style “BG_Keywords”). Twin-Chain Networks, liquid-liquid phase separation, Confocal Microscopy, gel roughness, cleaning, particulate matter

Additional Figures.

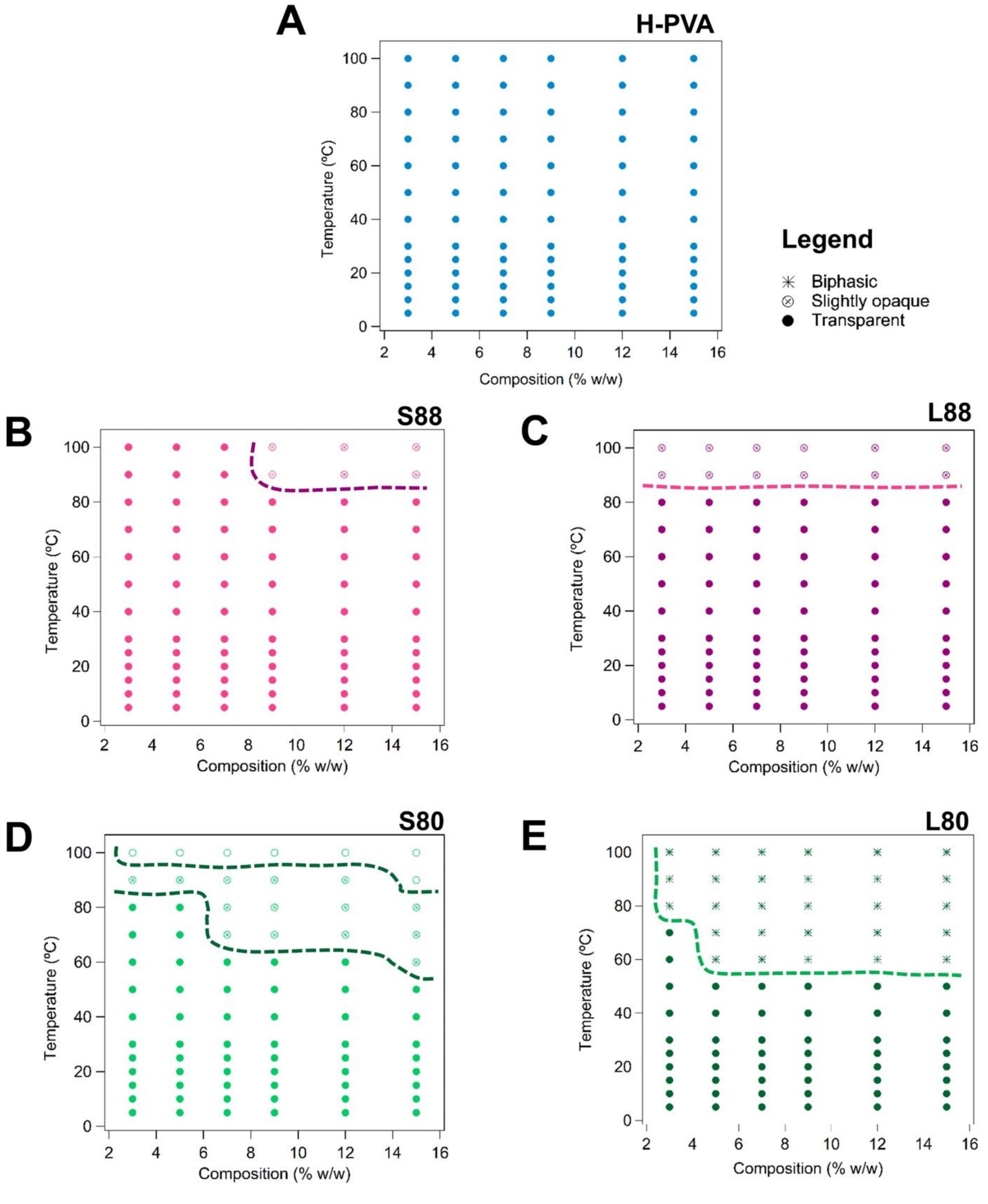

Figure S1. Phase-behavior of H-PVA, S88, L88, S80 and L80 in aqueous solution. The dashed lines indicate the different phases of the diagram and have been included to enhance the image readability.

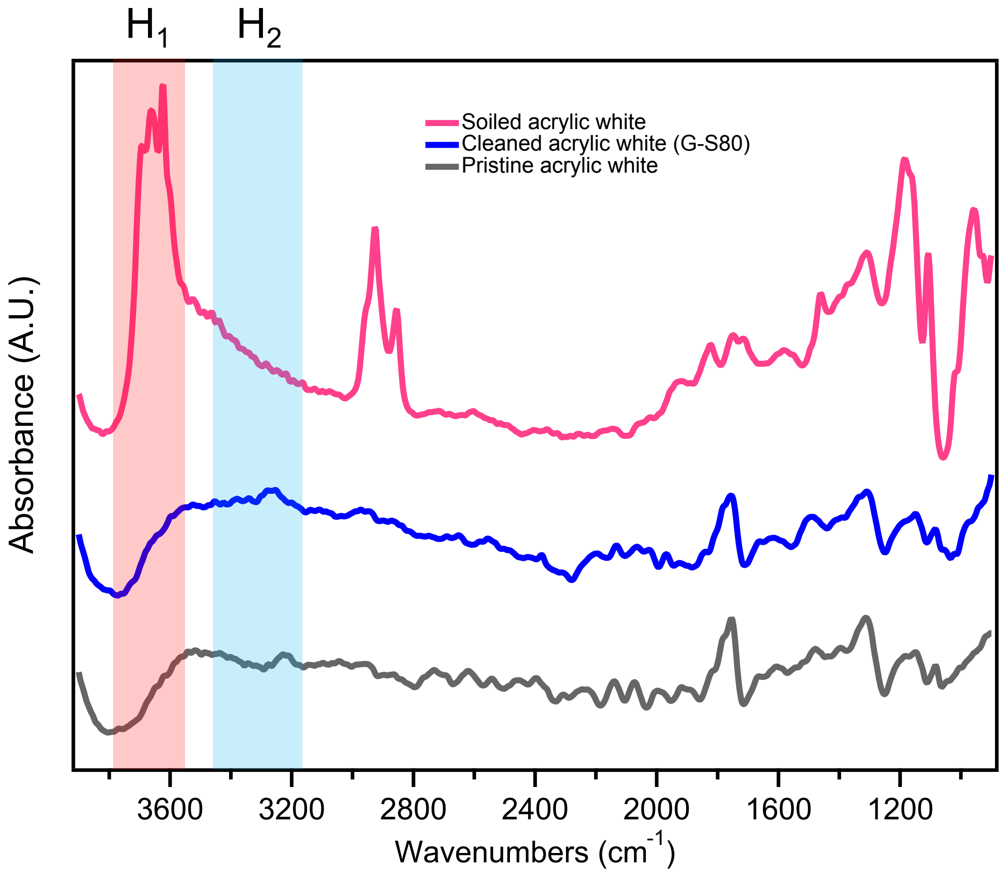

Figure S2. Infrared reflectance spectra corresponding to representative pixels (5.5 × 5.5 μm²) obtained from FTIR 2D imaging of the white acrylic mock-up (soiled, cleaned with gel formulation G-S80, and pristine). (H_1) Infrared range of hydroxyl stretching bands of kaolin (3725–3595 cm^{-1}), used for FTIR 2D mapping (Fig. 7); (H_2) Infrared range of hydroxyl stretching bands of PVA (3440–3180 cm^{-1}), used for FTIR 2D mapping (Fig. S3).

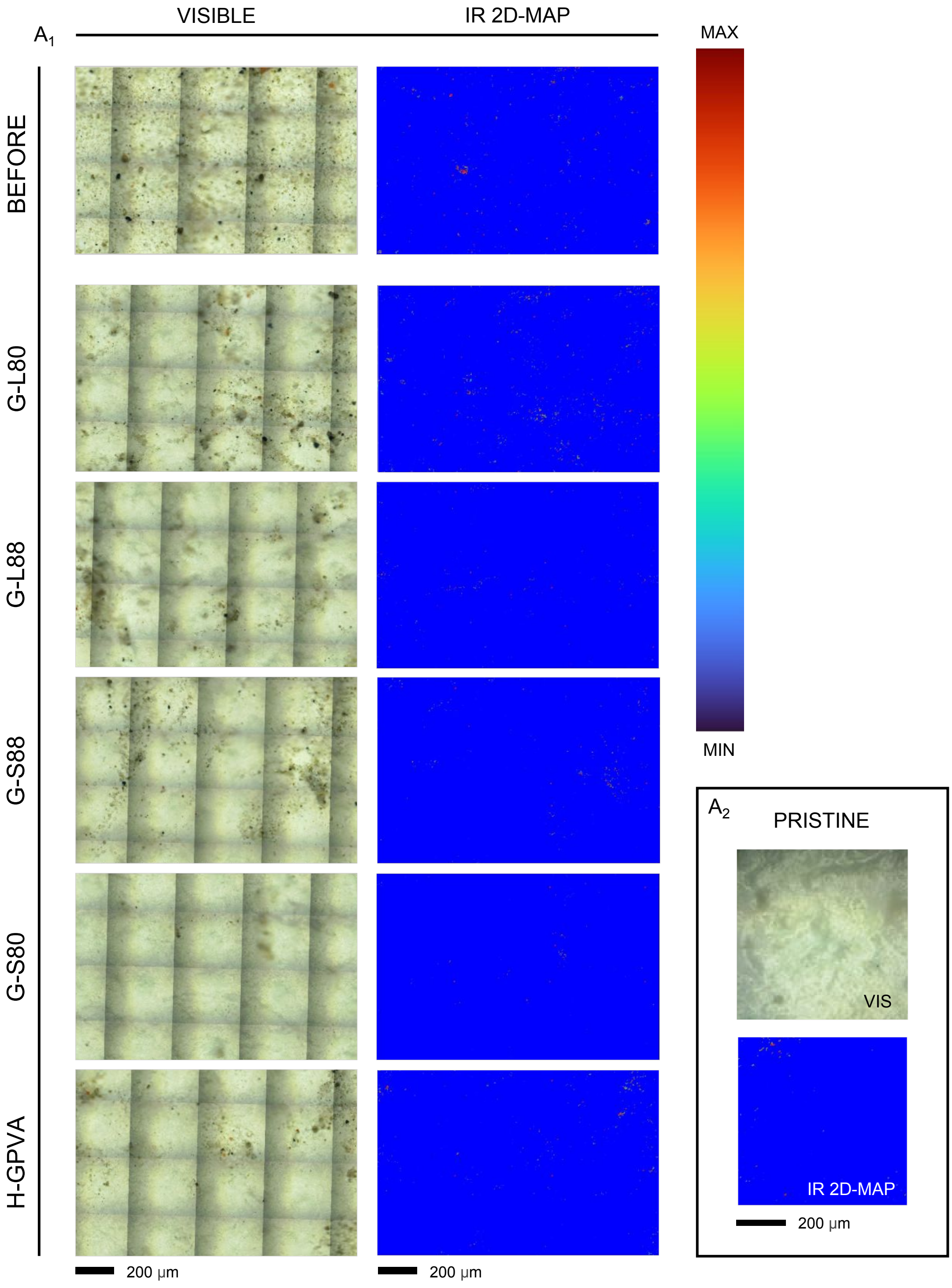

Figure S3. FTIR 2D imaging of the white acrylic mock-up: pristine state (A_2 panel), soiled (BEFORE), and after one cleaning cycle (10 min without rinsing) using G-S80, G-S88, G-L88, G-L80 and H-GPVA systems containing 5% wt TAC (A_1 panel). Adjacent to each visible map, the corresponding 2D FTIR map illustrates the intensity of the hydroxyl stretching bands of PVA (3440–3180 cm^{-1}).

Additional Tables.

Table S1. Slope values (1/λ) of curves shown in the chord analysis plot (fig. 3 A).

**Sample**	**1/λ**
**G-HPVA**	0.375 ± 0.007
**G-S88**	0.306 ± 0.010
**G-L88**	0.312 ± 0.005
**G-S80**	0.241 ± 0.008
**G-L80**	0.082 ± 0.004
